# Demographic and territorial characteristics of COVID-19 cases and excess mortality in the European Union during the first wave

**DOI:** 10.1007/s12546-021-09263-3

**Published:** 2021-05-29

**Authors:** Anne Goujon, Fabrizio Natale, Daniela Ghio, Alessandra Conte

**Affiliations:** European Commission Joint Research Centre, Ispra, Italy

**Keywords:** Age, Gender, Territory, COVID-19 first wave, Europe, Basic reproduction number, Excess mortality

## Abstract

This article explores for a large number of countries in the European Union (plus the United Kingdom) the main demographic differentials in positive tested COVID-19 cases and excess mortality during the first wave in 2020, accounting for differences at territorial level, where population density and size play a main role in the diffusion and effects of the disease in terms of morbidity and mortality. This knowledge complements and refines the epidemiological information about the spread and impact of the virus. For this analysis, we rely on the descriptive exploration of (1) data from The European Surveillance System (TESSy) database developed at the European Centre for Disease Prevention and Control (ECDC) on the number of cases and fatality rates and (2) of weekly mortality data collected by Eurostat. The analysis at territorial level studies the changes in R0—the basic reproduction number—and median excess mortality, across territories with different levels of urbanization. The unique findings of this study encompassing most European Union Member States confirm and define the demographic and territorial differential impacts in terms of infections and fatalities during the first wave of the pandemic in 2020. The information is important for stakeholders at European Union, national and sub-national levels in charge of designing containment measures for COVID-19 and adaptation policies for the future by anticipating the rebound for certain segments of the population with differential medical and economic needs.

## Introduction

Science is still unclear about the overall health impact and fate of the COVID-19 pandemic, which has challenged health systems and economies globally. Within the uncertainties surrounding the spread of the pandemic, demographers have been contributing to research by assessing how the virus had an uneven impact on the population, looking at several characteristics, such as age, gender, socio-economic characteristics, and place of residence. At the beginning of the pandemic, the literature focused mainly on the age structure of cases and fatalities, with the obvious observation that the elderly population was the most affected. Natale et al. (2020a) when comparing cases and fatalities of COVID-19 by age and regions in some of the most affected European countries, did not find significant differences between countries in *relative terms*, but showed that the age structure of population*s* determines the severity of COVID-19. Other studies came to similar conclusions (Dowd et al., 2020; Dudel et al., 2020). The prevalence of comorbidities along age, also in relation to socio-economic factors is critical for apprehending the impact of COVID-19 on mortality across and within countries. Old age has been identified as a risk factor in the disease, which has mostly to do with a higher frailty for the prevalence of chronic illnesses (Verity, 2020).

A further factor of heterogeneity in mortality due to COVID-19 was found to be gender where large sex differences exist with men having higher risk compared to women (Caramelo et al. 2020; Directorate-General Research & Innovation, 2020; Dowd et al., 2020). The female advantage might have to do with sex hormones like testosterone and oestrogen that seems to be key in adapting the body’s immune response (Zeng et al. 2020) and/or to genetics and the presence of two X-chromosomes, and/or to differences in the presence of intestinal bacteria (Directorate-General Research & Innovation, 2020). The role of gender-specific behavioural factors are more contested, such as smoking that could influence the higher prevalence of risk factors in men compared to women e.g. diabetes, hypertension and cardio-vascular diseases (Lippi & Henry, 2020; Patanavanich & Glantz, 2020; Changeux et al. 2020). Qian et al. (2020) noted that sex-differences in infectious diseases would require further epidemiological and biological investigations to design effective interventions. Also, more gender-differentiated analysis should be considered since the burden from COVID-19 of men and women is different, with especially increased inequities in terms of wellbeing and economic resilience to the disadvantage of women (Editorial The Lancet, 2020).

Place of residence is yet another factor of demographic heterogeneity, after age and sex. While density dependence^1^ has been shown to have a greater influence on mortality for nonhuman hosts such as animals and insects (Greenhalgh, 1992), the fact that the spread of COVID-19 (like that of Middle East Respiratory Syndrome (MERS) and Severe Acute Respiratory Syndrome (SARS)) occurs between close contacts via aerosols and droplets (Peeri, 2020) renders the study of place of residence (urban/rural) and population density highly relevant (see also Rocklöv & Sjödin, 2020). Population density and degree of urbanization have been shown to have a substantial impact on viral reproduction in the case of COVID-19—similarly to other viruses (Fang et al., 2009).

This article is organized around two main research questions: We first analyse the combined effect of gender and age in the absolute and relative number of cases and excess mortality to see whether the female advantage is visible at all ages, over the European Union. In a further step, we focus on the territorial differentials amidst all European Union regions^2^ looking at the spread of the virus across different levels of urbanization (rural, intermediate and urban) to study whether the urban disadvantage was present everywhere and throughout time. The research presented here has two main strengths. First, it systematically includes all European countries and territories (within the limit of data availability). Second, we make use of a unique database, The European Surveillance System (TESSy) data developed at the European Centre for Disease Prevention and Control (ECDC) that collects around 30 variables for each individual positive tested COVID-19 case reported by the European Union Member States with full epidemiological detail and the main demographic characteristics of individuals. This analysis takes place during the first wave of spread of COVID-19, around February to July 2020. While TESSy is the most harmonised, complete, and up-to-date dataset across the European Union for the first wave of the pandemic and at the time of the analysis, the results were still to be interpreted with caution due to data collection limitations: there have been differences across countries in case and death counting procedures, testing strategies (selection criteria, number and type of tests) and laboratory capacity that influence the results. To circumvent the issues with the TESSy database and validate our results, we estimated COVID-19 related excess mortality due to COVID-19 during the first wave using datasets available from EUROSTAT on the weekly evolution of the deaths in the European Union, available by age, sex and region.

When revising this article, at the beginning of 2021, the second wave was peaking in most European countries. There are still many unknowns about an evolving epidemic, and this is the reason for concentrating the research on the first wave, which ended during the summer of 2020, for which data are more complete and results have been confirmed. The findings regarding the demographic characteristics of the virus impact are important to inform further effective monitoring, containment, adaptation and recovery strategies (Nickbakhsh et al., 2020) which could be differentiated between distinctive populations.

After this introduction, we present in Sect. 1 the data and methods used in the analysis. Section 2 looks at the age and sex differentials while Sect. 3 concentrates on the urban–rural gradient in COVID-19 basic reproduction number and excess mortality. The results are discussed in the last section.

## Data and methods

### The European surveillance system database

The analysis developed in this paper relies mainly on two datasets. The first is part of the European Surveillance System (TESSy) database of the European Centre for Disease Prevention and Control (ECDC), a unique and most exhaustive database at European level. Our sample included detailed information on 643,916 positive cases as of July 3, 2020. There were noticeable differences in data provision between countries, reflecting differences in the epidemic phase and testing procedures^3^ adopted during the periods of data collection, as shown from Table 1, which provides an overview of the sample composition. For this reason, we filtered out the following countries for which the share of cases with detailed age and gender information was below 50% of the total cases reported internationally (as provided by ECDC): Belgium, Bulgaria, Croatia, Greece, Poland, Romania and the United Kingdom. As mentioned before, limitations might derive from the lack of harmonisation in data collection procedures among different institutions, with different methodologies and across different organizations. Therefore, the availability of data on the virus spread is not homogeneous in all regions analysed, depending on the number of tests carried out in each place.

**Table 1:** Coverage of ECDC data. *Source*: ECDC TESSy (July 3, 2020)

Country	Start date in 2020	End date in 2020	Share in total cases reported by ECDC of:		Total cases in TESSy	Total cases reported by ECDC
			TESSy cases with information on age and gender (%)	TESSy cases with information on place of infection or residence (%)		
Austria	22/02	03/06	91	91	16,705	18,269
Belgium	01/02	15/04	23	23	14,438	62,016
Bulgaria	08/03	11/03	0.1		6	5,740
Croatia	26/02	27/03	2	2	74	3,151
Cyprus	09/03	28/05	94		941	1,003
Czechia	02/03	31/05	72		9,038	12,515
Denmark	26/02	03/06	92	92	11,853	12,832
Estonia	27/02	30/05	94	94	1,869	1,993
Finland	28/01	03/06	96	96	6,952	7,253
France	23/01	19/03	5		10,072	166,960
Germany	28/01	02/06	93	92	182,376	196,554
Greece	26/02	12/04			2,141	3,519
Hungary	04/03	24/05	90		3,755	4,183
Iceland	28/02	12/05	97	96	1,801	1,863
Ireland	02/03	31/05	99	99	25,257	25,527
Italy	29/01	27/04	80	66	193,961	241,611
Latvia	03/03	31/05	95		1,065	1,124
Lithuania	28/02	04/06	90	90	1,656	1,836
Luxembourg	01/03	25/05	88		3,968	4,522
Malta	06/03	28/05	93		629	672
Netherlands	27/01	02/06	93		47,005	50,566
Norway	21/02	03/06	95		8,477	8,895
Poland	04/03	03/06	26		9,483	35,950
Portugal	21/02	31/05	79	79	34,542	43,897
Romania	01/03	22/05	41		11,790	28,973
Slovakia	06/03	31/05	85		1,505	1,764
Sweden	04/02	04/06	58	58	41,659	71,419
United Kingdom	29/01	10/04	0.1		178	285,416

Countries for which the share of cases with information on age and gender is below 50% are excluded from our sample. Additional details on completeness of datasets are available from https://covid19-surveillance-report.ecdc.europa.eu/#5_tessy_data_quality. Data for France on place of infection and residence were not available in TESSy and were downloaded from https://geodes.santepubliquefrance.fr/ (July 03, 2020) – not shown in the table

Data for France at regional level were not available in ECDC-TESSy and were downloaded from Géodes (Géo données en Santé Publique) (July 3, 2020). Again here and similarly to the issues pointed out for the TESSy database, the reporting system in Géodes is not exhaustive and the number of reporting establishments varies over time.

### The Eurostat dataset on weekly deaths

To complement the ECDC dataset, and validate our findings regarding casualties, we rely on Eurostat datasets to calculate excess mortality. The datasets are part of a special data collection on weekly basis to inform public users and support policy makers dealing with the definition of strategies to cope with the COVID-19 crisis. This exceptional (and temporary) data collection consists of the number of weekly deaths disaggregated by gender and 5-year age groups at NUTS3 level with time series starting in some cases in 2000, transmitted by the National Statistical Institutes to Eurostat on a voluntary basis (see Table 2). Weeks are classified according to the ISO8601 classification. Deaths are recorded by the date of occurrence of the event (except in Latvia and the United Kingdom, where records indicate the date of death registration) referring the deceased individual to his/her usual residence (by region).^4^ The coverage of the database is substantial since overall, European Union Member States record around 95% coverage by weekly period.

**Table 2:** Coverage of data on weekly mortality with breakdown by age, gender and NUTS3 region. *Source*: Eurostat (demo_r_mweek3) downloaded on 04/12/2020

Country	Start date	End date	Number of NUTS3 regions
Austria	03/01/2000	09/11/2020	35
Belgium	03/01/2000	16/11/2020	44
Bulgaria	04/01/2010	23/11/2020	28
Cyprus	05/01/2015	16/11/2020	1
Czechia	03/01/2005	26/10/2020	14
Denmark	08/01/2007	23/11/2020	11
Estonia	03/01/2000	30/12/2019	5
Greece	05/01/2015	31/08/2020	53
Spain	03/01/2000	23/11/2020	59
Finland	03/01/2000	23/11/2020	19
France	07/01/2013	16/11/2020	101
Hungary	03/01/2000	09/11/2020	21
Italy	12/01/2015	31/08/2020	110
Lithuania	03/01/2000	23/11/2020	10
Luxembourg	03/01/2000	02/11/2020	1
Latvia	03/01/2000	30/11/2020	6
Netherlands	04/01/2016	16/11/2020	40
Poland	03/01/2000	02/11/2020	73
Portugal	03/01/2000	09/11/2020	26
Romania	05/01/2015	28/09/2020	42
Sweden	03/01/2000	23/11/2020	21
Slovakia	03/01/2000	02/11/2020	8
United Kingdom	05/01/2015	23/11/2020	179

To be consistent with the analysis of cases from ECDC we only use data until 03/07/2020

### R0 calculation method

A key indicator to monitor the spread of a disease and to design control measures and vaccination campaigns is the basic reproduction number R0. R0 by itself does not tell how fast the disease is spreading but rather how difficult it is to contain it. The value of R0 is affected by the infectiousness of the pathogen, the contacts’ intensity and the infectivity of individuals. We consider R0 rather than Rt—the effective reproduction number, calculated as the number of cases in the current state of a population, which does not have to be the uninfected state—since the analysis is carried out at the early stages of the epidemic.

Taking as constant infectiousness and infectivity, we can assume that changes in R0 across territories are depending on the effect of population density on the intensity of contacts. The rationale is that higher population density which is reflected in the definition of the urban typology may imply a higher R0 by increasing the probability of contacts between individuals.

One of the most common approaches to calculate the R0 especially in the initial phases of the epidemic is to examine the observed exponential epidemic growth rate of cases (Wallinga & Lipsitch, 2007).

The mathematical relation between R0 and the exponential growth rate is described by the following equation:

$$R = 1/M\left( { - r} \right){ }$$

where *M* is the moment generating function of the (discretized) generation time distribution and *r* is the exponential growth rate, in our case estimated using a Poisson distribution.

The generation time distribution refers to the time lag between infection in a primary case and a secondary case. Since this is often not available, as a substitute we use the serial interval distribution which is defined as the period between the appearance of symptoms in the infector and in the infected. For COVID-19 we rely on values reported in Zhanwei et al*.* (2020) which have been calculated examining 468 confirmed cases of COVID-19 in China as of February 8, 2020 (mean interval of 3.96 days, standard deviation of 4.75 days).

For the calculation we use the package R0 (Obadia et al., 2012). After constructing the specific epidemic curves for each territory (NUTS3), we iterate the calculation of R0 for each curve. Finally, we represent the results by different groupings of rural–urban typologies.

Another important aspect to consider in the calculation, is that R0 changes depending on the phase of the epidemic, starting from the highest value during the exponential growth phase and gradually approaching the target of below 1 which is signalling that the epidemic is dying out, either autonomously or under the effect of containment measures. We want to test if the differences in R0 between territories are persisting independently from the phase of the epidemic. For this we repeat the calculation of R0 considering increasing temporal windows of 5 to 40 days, and including in each calculation all the territories where the epidemic has been lasting at least for the duration of each temporal window.

### Method for calculating COVID-19 excess mortality

The long time series of deaths across the European Member States is used for the statistical modelling of excess mortality. We adopt a generalized additive model (GAM). The modelled baseline is fitted independently for each geographical unit (NUTS3 regions) and each age-gender group. The main advantage of the GAM model is the presence of additional controls, such as a seasonal component to account for the increase in mortality during winter months, and time-trend linearity to account for long term changes in mortality reflecting population ageing.

**Fig. 1:**
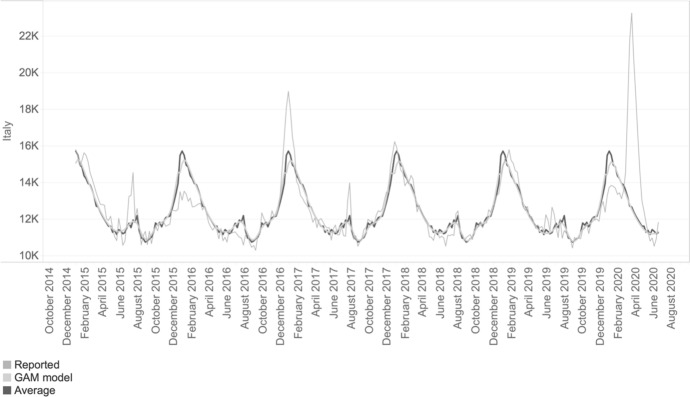
Comparison of the weekly reported number of deaths to estimated trends using a simple average and GAM model, Italy (January 2015 to June 2020). *Source*: Authors' calculations based on Eurostat data

The model is fitted independently for each geographical unit and each age-gender group using as training set all the available historical data before 2020. By excluding the period before the spread of COVID-19 this estimated baseline represents the expected level of mortality, considering the specific characteristics of populations by age and gender living in each NUTS3 region. Since we exclude from the training set the COVID-19 periods we set up a baseline before the pandemic. In this baseline most of the seasonal variations in weekly mortality are observed in correspondence of the influenza outbreak with peaks normally around January and February. Additionally, and for sensitivity check purposes, we compute a second baseline considering the weekly averages of deaths for each region age and gender (see Fig. 1 as an example for Italy of the resulting baselines of the GAM and average models compared to the reported data).

As shown by the example (and same for all countries), the GAM model leads to comparable results to the simpler average method. Yet, the main difference consists in better smoothing by the GAM model when compared with the average method, as displayed in Fig. 1 for the most recent influenza outbreaks. This better fitting of the GAM method justifies our choice for the subsequent analyses on excess mortality.

To calculate the excess mortality for each age and gender group and region, we subtract the reported deaths in 2020 from the GAM estimated baseline for all occurrences exceeding the upper 95% confidence intervals of the model results and divide this difference by the baseline. These values are finally summarised by regions grouped by degree of urbanisation focusing on the period February 1st to June 30th, 2020 to explore the mortality impact during to the first wave of the pandemic.

## Gender differentials by age

**Fig. 2:**
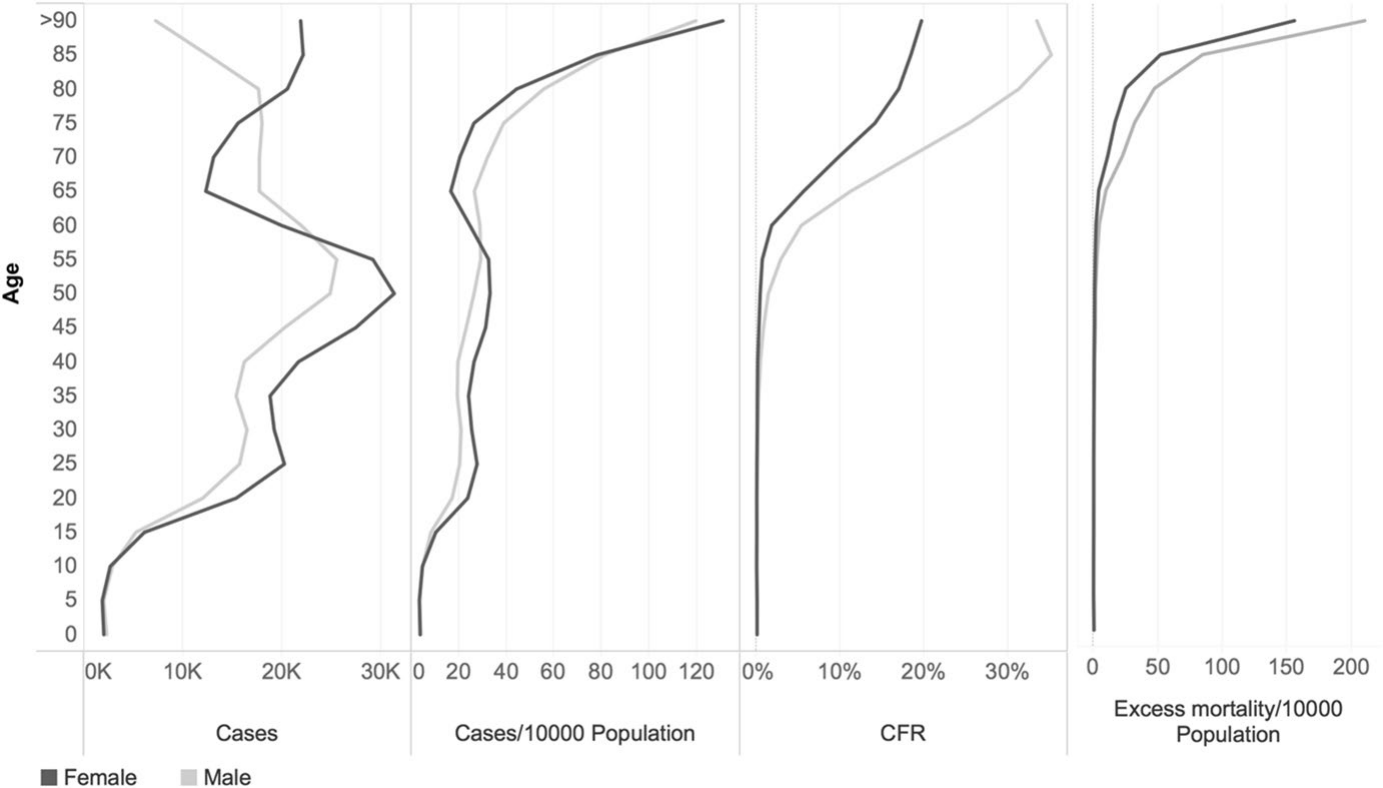
(A–D) Distribution of cases in absolute numbers (A), cases per 10,000 people (B), case fatality rates (CFR) (C) and excess mortality per 10,000 people (D) by age and gender, selected countries. *Source*: ECDC TESSy and Geodes—France (July 03, 2020) for cases and CFR; Eurostat for excess mortality

**Fig. 3:**
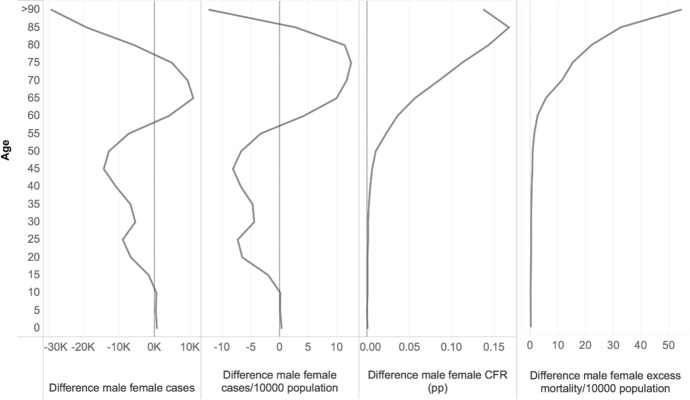
(A–D) Distribution of the difference between male and female cases in absolute numbers (A), cases per 10,000 people (B), case fatality rates (CFR) (C) and excess mortality per 10,000 people by age (D), selected countries. *Source*: ECDC TESSy and Geodes—France (July 03, 2020) for cases and CFR; Eurostat for excess mortality

COVID-19 is incapable of discrimination. However, the consequences of the virus are not the same for women and men, both in terms of positive tested COVID-19 cases and excess mortality as shown in Figs. 2A and 3A by age for the whole sample.

Overall, the cumulative number of diagnosed positive cases was particularly large among the population between 35 and 65 years of age. This group which consists mostly of a population of working-age represented 50% of confirmed positive cases, indicating that the infection did not only affect the elderly (20% of all cases belonged to the 75 + age-group).

Looking at gender differences in COVID-19 positive tested cases, we observe that more cases were reported among men aged 55 to 80 years old compared to women, while higher numbers of positive cases were reported among women aged 15 to 55 years and above 80 years of age compared to men. The male disadvantage was particularly pronounced for the age group 65–70. For instance, in Italy and Belgium, men aged between 60 and 80 years of age were respectively 1.5 and 1.4 more likely to be reported as positive as women. The higher number of cases for men could be linked to the fact that testing in the early phases of the pandemic was primarily performed on critical and severe cases. Since elderly men face more serious consequences than women, they could, as a result, have been more likely to be tested. For some other countries like Germany and Portugal, the male to female cases ratio was close to 1 around the 60–75 age groups.

**Fig. 4:**
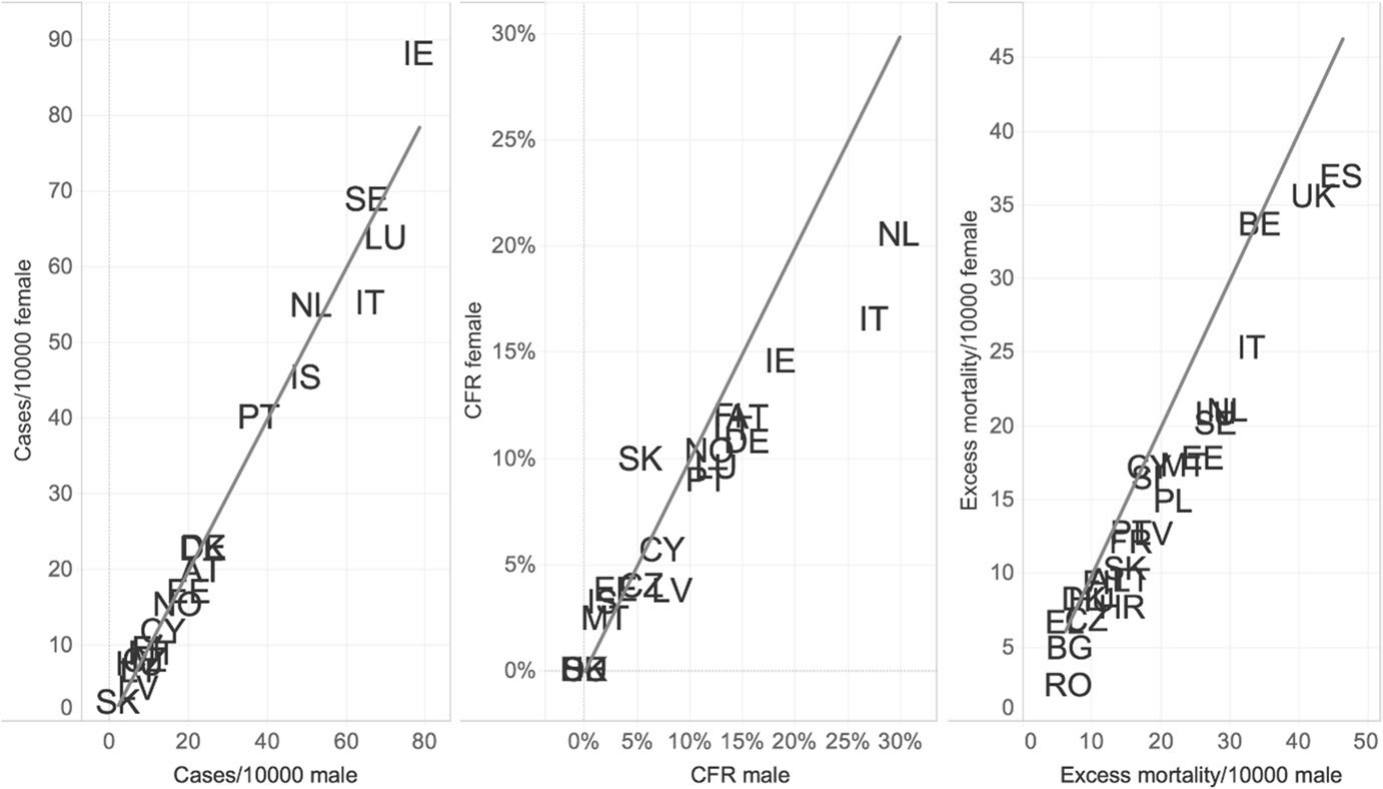
Comparison between the cases per 10,000 people, case fatality rates and excess mortality per 10,000 people for men and women above 60 years of age by country. *Source*: ECDC TESSy and Geodes—France (July 03, 2020) for cases and CFR, Eurostat for mortality. *Note*: Values below the diagonal indicate higher incidence for men than for women. The country codes are the following: AT-Austria, BE-Belgium, BG-Bulgaria, CR-Croatia, CY-Cyprus, CZ-Czechia, DK-Denmark, EE-Estonia, FI-Finland, FR-France, DE-Germany, EI-Greece, HU-Hungary, IS-Iceland, IE-Ireland, IT-Italy, LV-Latvia, LT-Lithuania, LU-Luxembourg, MT-Malta, NL-Netherlands, NO-Norway, PL-Poland, PT-Portugal, RO-Romania, SK-Slovakia, ES-Spain, SE-Sweden, UK-United Kingdom

When related to the total population by age groups (Figs. 2B and 3B), the proportion of patients diagnosed with COVID-19 was higher among women under the age of 50; in the sample, there were indeed 10 infected women against 8 infected men for every 10,000 persons below the age of 20 years living in the countries. The proportion was about the same for men and women around the age of 50–55 years; yet, from the age of 58 onward, the male prevalence among notified positive cases became evident. The comparison across countries reported in our sample (Fig. 3) confirms that positive cases among the male population above the age of 60 years were overrepresented in relative terms compared to female ones in many countries. The diagonal of Fig. 4 indicates the equal distribution between gender; values below the diagonal show the higher prevalence of cases among men. At a higher number of cases per 10,000 population, the male disadvantage seems also larger compared to countries with lower relative numbers, except for Ireland.

Since the number of positive cases in the dataset corresponded largely to the number of symptomatic individuals who were present at health facilities, this gives a broad indication that COVID-19 related fatality risks are of particular concern for men. As Fig. 2C shows, the case fatality rate (CFR—the proportion of deaths from COVID-19 compared to the total number of positive tested cases) confirms the male disadvantage: the CFR was higher among men for all age groups. Overall, the male mortality disadvantage peaked at the age of 85 when the CFR for men was 9.7% higher than that for women (24.1% and 14.4% respectively). Figure 4 shows that COVID-19 related fatalities were higher among men aged 60 years and over in all countries, with the exception of Cyprus and Slovakia. Gender differences were particularly substantial in the Netherlands, where the CFR for men aged 60 + was 8 percentage points higher than for women (25% and 17% respectively), similar to Italy, where the male CFR was 22 percent against 15% for women.

Figure 2D makes evident how gender differences persist in excess mortality across age groups, therefore confirming the findings derived from CFR. Goldstein and Lee (2020) have demonstrated that the age pattern of COVID-19 follows a similar pathway to all diseases and causes of deaths, shaping as a Gompertz hazard curve, with an exponential increase by age group. Similarities in the shape allow the comparison between COVID-19 related rates and general mortality trends. Focusing on the first wave as a whole lowers the impacts of differences in data recording across countries, particularly during the first few weeks of the pandemic (Banerjee et al., 2020). Using the GAM model, we estimate that the excess deaths per 10,000 people would range from 5 deaths among men in the 60–64 age group to 210 deaths above the age of 90 years, whereas for women, the excess deaths were 2 for women aged 60–64 years and 155 for women above the age of 90 years.

Gender differentials are displayed by age group in Fig. 3D, while Fig. 4 plots national differentials between male and female excess mortality per 10,000 people. Male disadvantage is confirmed in the vast majority of European countries, when only in three countries (Belgium, Denmark and Greece) gender differences were minor (countries appear on the diagonal). Previous studies have documented how excess mortality due to COVID-19 varies across countries (e.g., Félix-Cardoso et al. (2020) compute variations from 10.6% in Portugal to 98.5% in Italy). In line with these results, we find that Spain accounts for the highest estimated excess mortality by gender (36.9 extra deaths per 10,000 women and 46.3 for men, which implies a ratio of 9.4), followed by the United Kingdom (35.5 for women and 42.2 for men, and a ratio of 6.7), Belgium (33.6 for women and 34.4 for men, and a ratio of 0.7) and Italy (25.2 for women and 33.1 for men, and a ratio of 7.9). The lowest estimated excess mortality refers to Romania (2.4 for women and 6.7 for men, and a ratio of 4.2) and Bulgaria (4.9 for women and 6.9 for men, and a ratio of 2).

**Fig. 5:**
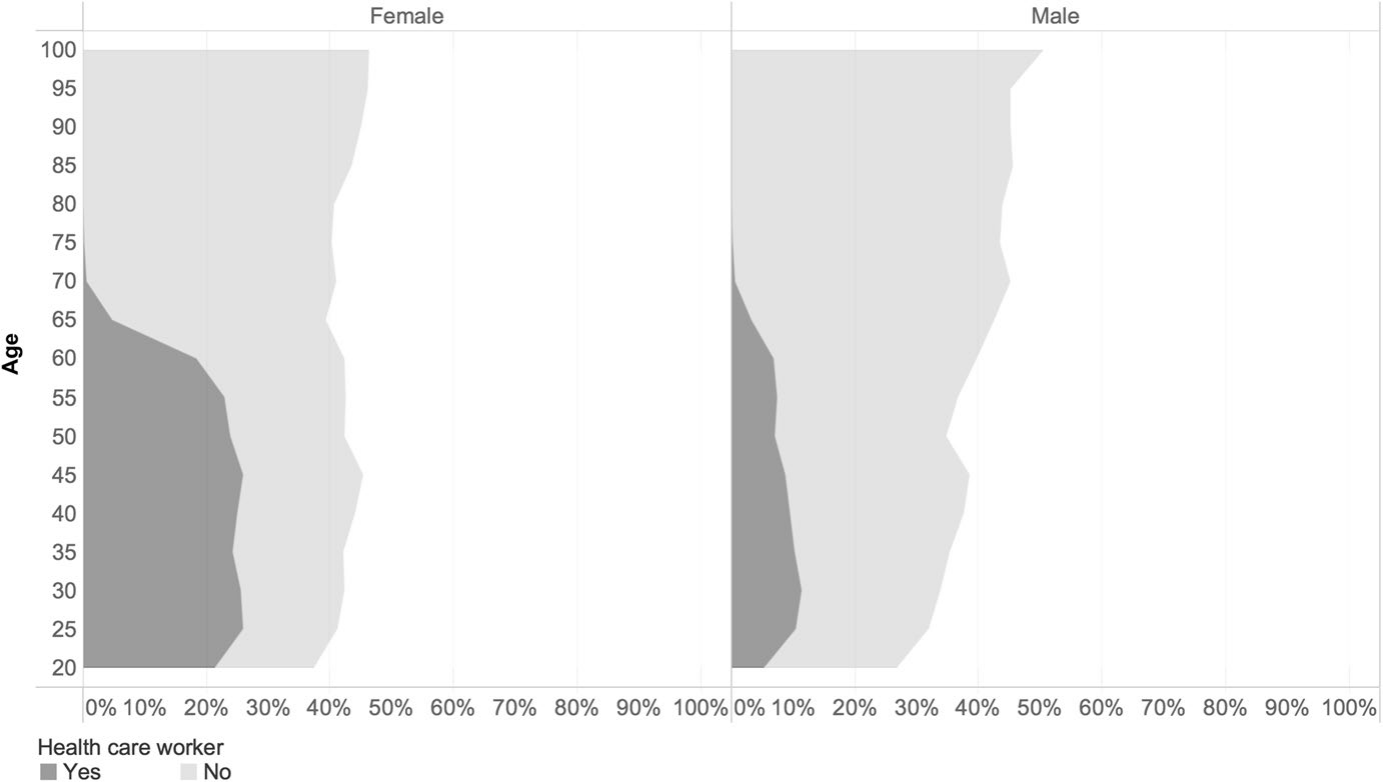
Percentage of healthcare workers among cases by age and gender. *Source*: ECDC TESSy and Geodes—France (July 03, 2020). *Note*: Overall healthcare workers represent 6.6% (25,963) of total cases

Our results suggest that there is an age and gender divide in COVID-19 cases and fatality rates, which does not necessarily reflect and cannot be explained exhaustively by the population structure. This is not unprecedented as seen from other epidemics. During the Ebola epidemic, women were more likely to be infected by the virus, given their role as caregivers within families and their overrepresentation as health-care workers (Davies & Bennett, 2016). Chinese official sources reported the higher risk that female health workers incurred in Hubei province, where they represented more than 90% of health-care workers (Boniol et al., 2019). In some European Member States, there has been increasing evidence that outbreaks in elderly homes have spilled over via the health care personnel, which is a sector that commonly reports a majority of female employees.^5^ It is clear that such spill over could occur outside elderly homes, exposing patients assisted at home to become additional clusters of infections. The higher number of cases among women below the age of 55 in European Union Member States could stem in part from their over-representation in some front-line occupation, like in the health sector as well as from their higher care-giving responsibilities within the family compared to men (see Discussion). Figure 5 shows that the proportion of health care professionals among diagnosed cases was substantially larger for women than for men. However, the data have to be interpreted with caution considering that healthcare workers are much more likely to be tested.

The male disadvantage is evident in COVID-19 fatality and excess mortality. Although the lack of accuracy on individual clinical data availability limits empirical demonstrations, we can suggest that the higher fatality rates for men may derive from gender-based immunological differences (Chen, 2020), or from the association with comorbidities, including hypertension, cardiovascular diseases and drinking alcohol more commonly observed among men (Editorial the Lancet, 2020).

Previous outbreaks have shown the relevance of analysis of the role of women in society into the response efforts and definition of policy interventions. These results also confirm that the current pandemic is linked to the specific demographic characteristics of the population resulting in outcomes that affect the population in an uneven way. This evidence therefore has significant implications for the definition of a recovery strategy. The effectiveness of measures to gradually lift the lockdown and restarting the economy will also depend on the integration of the demographic profile into its strategy.^6^ Another aspect that will be important and that we tackle in the next section is that of the territorial specificities.

## Territorial differences

Urban areas, and especially large cities, have been severely affected by the rapid spread of the COVID-19 pandemic, recording the highest numbers of cases and deaths. Although the incidence of the virus at the beginning of the pandemic was significantly higher in densely populated regions, the pandemic slowly spread outside of these regions, with different health and economic implications for the affected areas (Cerqua & Letta, 2020). The evidence on the link between population density and virus spread has been rather inconclusive (Chowell, 2008; Garrett, 2007). For Li et al. (2018), the spread of outbreaks is related to population density only when appropriate density ranges, evenly distributed population, and the initial proportion of individuals most susceptible to new infections are considered. Chandra et al. (2013) found that in the case of the 1918–19 flu pandemic in India, there was a population density threshold—of 175 persons per square mile—above which containment policies were effective. Regarding the COVID-19 pandemic, the analyses by Stier et al. (2020) and Ribeiro et al. (2020), suggest that city density and size intensify the spread of the virus at the beginning of the pandemic; over time, however, the rate of growth in cases tends to decrease in large cities and increase in small towns. In contrast, Heroy (2020) found no evidence of the effect of the city size on cases from COVID-19 at the county level in the United States.

The empirical literature on the differences in the territorial spread of COVID-19 is growing fast, with some analyses considering multiple socioeconomic and demographic factors related to the urban organization, and with a primary focus on connectivity, occupations most susceptible to the transmission, and crowded housing conditions. Large cities are major hubs for transportation systems, which are facilitating the spread of the virus from urban centres to peripheries and across the country. Mazzoli et al. (2020) link the high prevalence of COVID-19 in sparsely populated areas in Spain with the frequency of weekend travel to and from Madrid. Also for Gerritse (2020), the use of public transportation is initially linked to increased virus transmission. Harris (2020) and Tian et al. (2020) describe how mobility patterns in New York City and several Chinese cities have led to crowding in public transportation, thus becoming a source of infection.

The significant territorial disparities in the industrial and occupational structure (Autor, 2019) seem to have played a decisive role in the spread of the virus. According to Almagro and Orane-Hutchinson (2020), the intensity of contacts in the so-called essential jobs is crucial to explain how the infection spread unevenly in New York City neighbourhoods. Chiou and Tucker (2020) analyse the role of the digital divide in the effectiveness of social distancing measures and find Internet access particularly important for wealthy areas where the large number of jobs that can be performed remotely are concentrated. The spread of the virus has also been analysed in differently populated areas in relation to the territorial economic models. According to Agnoletti et al. (2020), the presence of industrial and agro-industrial activities in Italy is correlated with the infection rate even after controlling for the demographic, economic and environmental characteristics of the provinces.

Finally, the empirical evidence has also focused on the characteristics of the housing units (Gormley et al., 2020), which have been described as more conducive to virus transmission when they were overcrowded^7^ (Natale et al., 2020b). In this line of analysis, family ties and intergenerational relationships are of relevance for virus transmission especially in environments where people tend to be in close contact and with family members from different generations living together (Bayer & Kuhn, 2020). Di Gialleonardo et al. (2020), with a cross-national analysis, show that family ties are a key variable in explaining the difference in the spread of the COVID-19 pandemic. Countries where family ties are important show higher numbers of infections and deaths. In contrast, the analysis by Belloc et al. (2020) across Italian regions indicates a negative correlation between case fatality rates and an index of vertical social interaction. Overall, transmission of COVID-19 has been documented to be higher within families than in other community contexts, with the results explained by individual, behavioural, and contextual factors (Merckx et al., 2020).

**Fig. 6:**
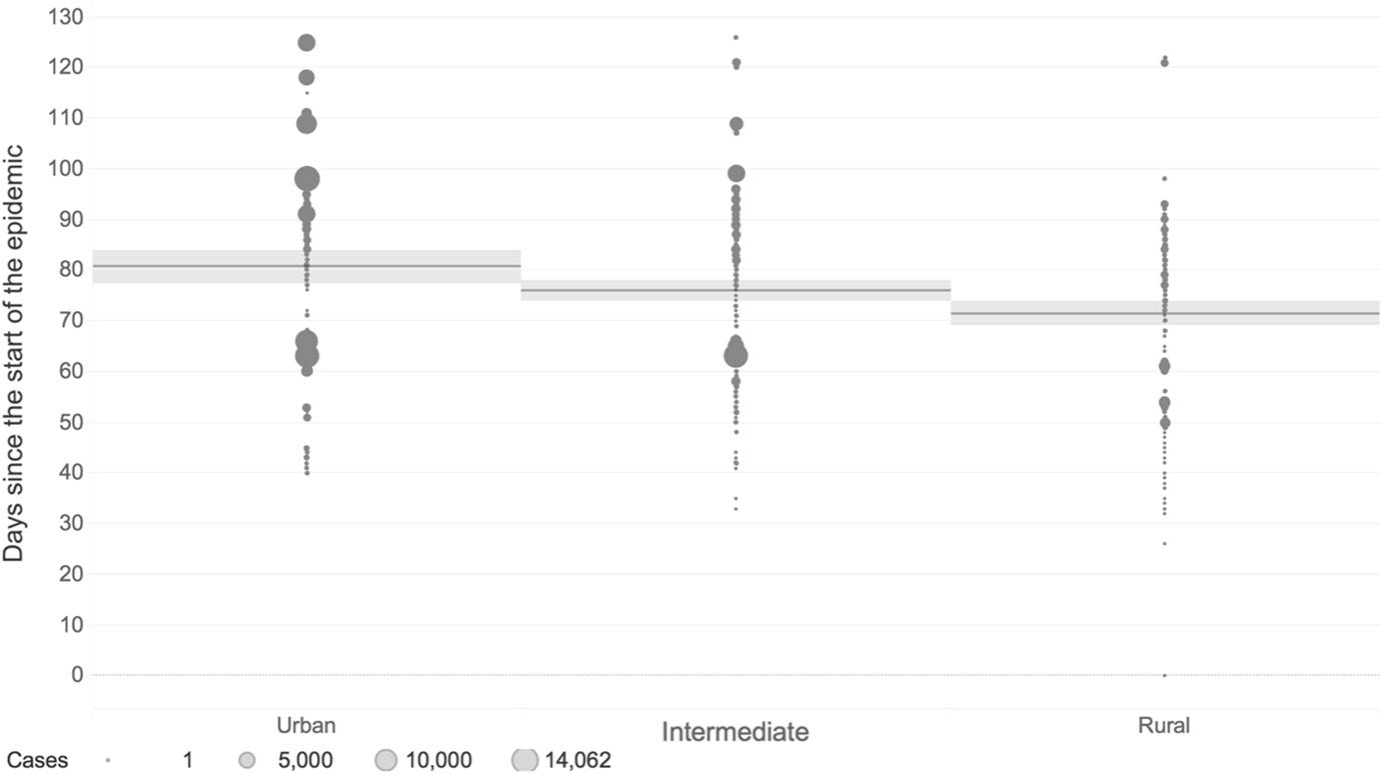
Duration of the pandemic in each region as the number of days between the 1st and the most recent reported case by urban–rural regional typology of European Union regions. *Source*: ECDC TESSy and Geodes—France (July 03, 2020). *Note*: Each circle is representing a region, the size is proportional to the number of cases and the horizontal line is the average with 95% confidence by the urban–rural regional typology

The duration of the pandemic in each region, as measured by the days between the first and last reported case during the first wave is illustrated in Fig. 6. The regions are clustered by three types of degree of urbanization.^8^ The sample consists of 187 urban regions, 374 intermediate regions, and 286 rural regions. Since the first COVID-19 case was detected, the pandemic has evolved more rapidly and with higher incidence in most urban regions, where a large proportion of the European population lives. According to Eurostat, in 2019, 40% of the European population lived in urban regions, 39% in intermediate regions, and 21% in rural regions. Virus spread declined along with population density, with intermediate and rural regions experiencing fewer cases during the first wave. However, intermediate and rural regions in Europe have not been immune to the risks associated with the spread of the virus, being particularly vulnerable due to population demographics, income levels, and limited access to medical resources.

**Fig. 7:**
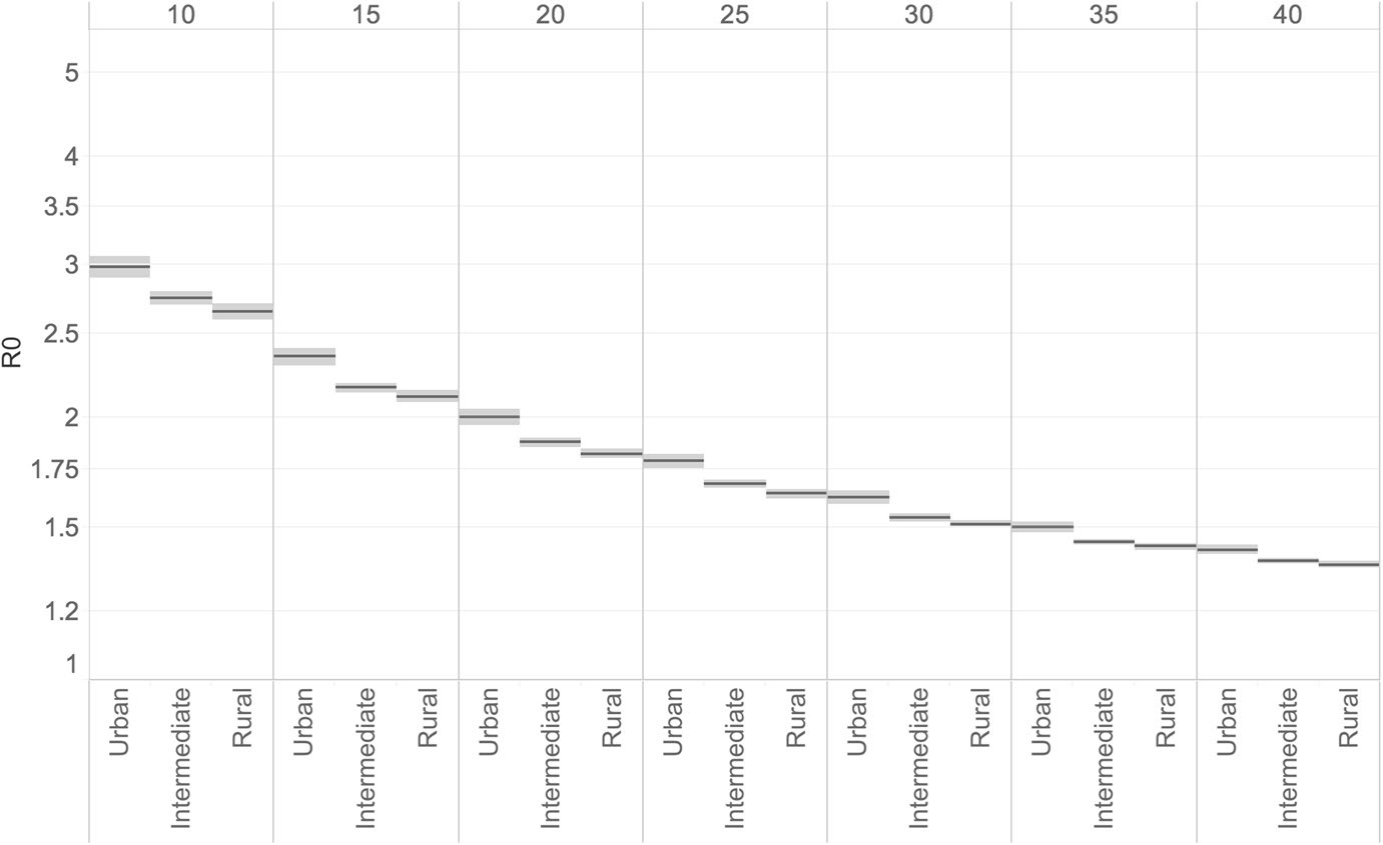
R0 by the urban–rural regional typology of European Union regions, by temporal window (10–40 days). *Source*: ECDC TESSy and Geodes—France (July 03, 2020). *Note*: The calculation is repeated considering increasing temporal windows (10–40 days) since the start of the epidemic. The horizontal line is the average of R0 with 95% confidence intervals by the urban–rural regional typology. Traditionally the OECD has classified TL3 regions as predominantly urban, intermediate, or predominantly rural regions (respectively 3_PU, 3_IN,3_PR). This typology is mainly based on population density in each local unit, combined with the existence of urban centres where at least one-quarter of the regional population reside

Figure 7 shows the territorial disparity in the virus contagiousness, as measured by the Basic Reproduction Number R0, corresponding to the average number of secondary infections of a case at the beginning of the epidemic. We track the number of cases through the daily R0 estimate to determine the different levels of infection by region. Differences in contagiousness based on reported cases appear to follow the territorial demarcation lines, with the virus affecting the most the larger populations in urban regions. For those living outside urban regions, the impact was smaller. In the first 10 days of the outbreak, the estimated R0 (with a 95% CI) was equal to 3 in urban regions, meaning that each new positive case of COVID-19 produced three new cases. The average for intermediate regions at the beginning of the epidemic was about 2.8 new cases per infected person, followed by rural regions with an average of about 2.6. During the first wave of the pandemic, R0 values—as well as the differences across regions in the index—generally declined; by day 40 of the outbreak, however, R0 values in the three regions were still above 1, which is the level above which the infection continues to spread.

**Fig. 8:**
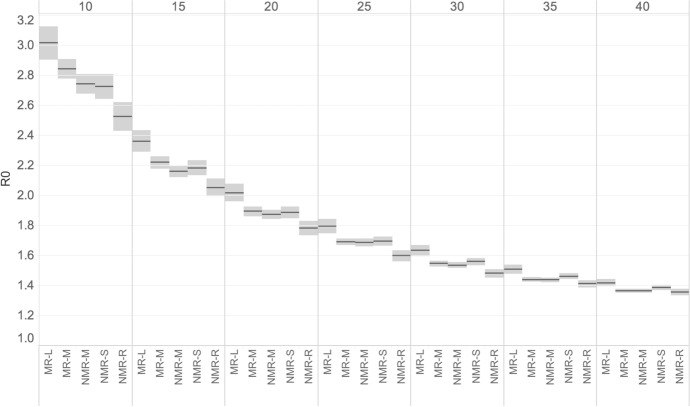
R0 by the OECD territorial grid (TL3), 5 classes of metropolitan and non-metropolitan regions for European Union regions and by temporal window (10–40 days). *Source*: ECDC TESSy and Geodes—France (July 03, 2020). *Note*: The calculation is repeated considering increasing temporal windows (10–40 days) since the start of the epidemic. The horizontal line is the average of R0 across all regions with 95% confidence intervals

Figure 8 shows the results of the sensitivity analysis on R0 based on the OECD classification at lower territorial levels (LT3) based on metropolitan and non-metropolitan population. In particular, Fig. 8 shows the results according to five different territorial classes that allow a clearer view of the course of the infection. Regions are classified according to their level of access to metropolitan areas and divided into "metropolitan" if more than half of their population lives in one or more functional urban areas (FUAs) of at least 250,000.^9^ In all cases, virus transmission is highly dependent on population size. This additional territorial classification supports the findings of a viral infection that in the first phase primarily affected more densely populated regions, as well as a progressive reduction in the number of infections as population size decreases. Over time, we also observe a decrease in disparities across regions. These results confirm previous analyses, including the work of Goujon et al. (2020) that was conducted looking at 9 spatial classes for counties in the US and based on data from USAfacts.org. Population size is a key determinant of the level of infection in the early phase of the pandemic, with regions with more than 1.5 million people experiencing the highest R0 values and less populated regions experiencing the lowest values. We observe, however, some exceptions to the general trend, with some regions classified as NMR-S—i.e., regions that do not have access to a metro and 50% of their population has access to a small or medium city (a FUA of more than 50 thousand and less than 250 thousand inhabitants) within a 60-min drive—showing higher R0s than more populated regions, which would require further investigation.

**Fig. 9:**
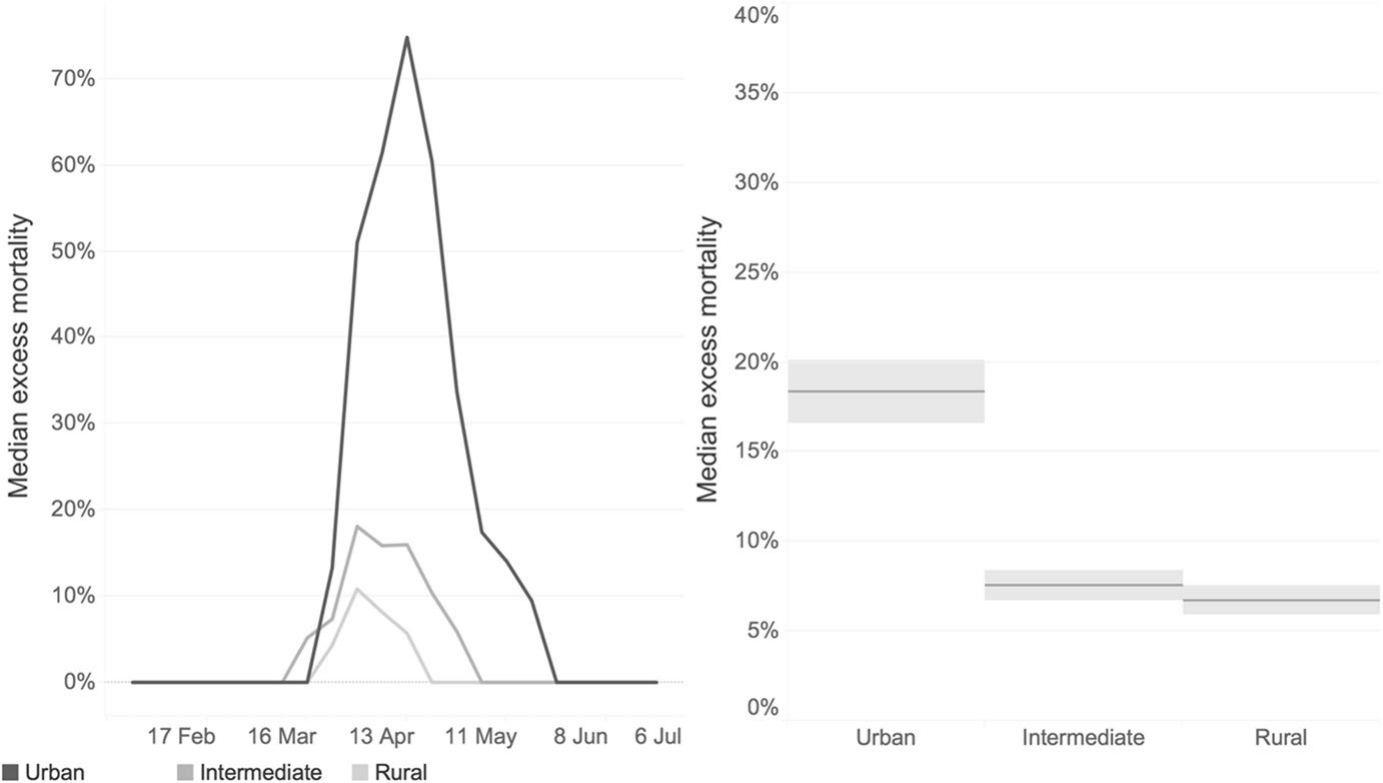
Median excess mortality across European Union regions, by week (left) and for the entire period (right, February-June 2020) by degree of urbanisation. *Source*: Authors' calculations based on Eurostat data

Overall, the dichotomy between urban and other degrees of urbanization in R0 are confirmed by the analysis based on excess mortality as shown in Fig. 9. The number of weekly deaths attributable to COVID-19 peaked in early April. During this peak, the median excess mortality in the case of urban regions was 75%, followed by the percentages recorded in intermediate (16%) and rural regions (6%). For the entire period analysed, excess mortality was about 18% for urban regions, 8% for intermediate regions, and 6% for the rural ones.

## Discussion and conclusion

While the gender difference in COVID-19 related cases and deaths among Europeans has been well documented (see for instance the meta-analysis in Pérez-López et al. (2020)), it is less clear how this interacts with age. Results from the analysis suggest that there was an age and gender divide in COVID-19 infection, fatality rates, and excess mortality during the first wave, which did not necessarily reflect the population structure, which has on the other hand be shown essential in explaining the distribution of cases and fatalities (Dowd et al., 2020). There are more reported cases of diagnosed women by COVID-19 compared to men in the population below the age of 60 years. One possible reason is that women represent a high percentage of workers in higher-contact employment such as in the health care sector and care facilities and are therefore at the forefront of the fight against COVID-19, or in frontline services work (such as teacher, service clerks, cashiers or cleaners). Moreover, for those working in health and care facilities they have a potentially higher chance of being tested. Tadiri et al. (2020) found a positive relationship between the gender inequality index (provided by the United Nations Development Programme) and the male to female ratio in reported cases of COVID-19, across 33 countries, which could be linked to the higher occupation opportunities of women in essential services in those countries. The higher probability of infection for women below the age of 60 years compared to men, does not however translate into higher mortality. A study in Sweden has found no evidence of a linkage between frontline worker occupations and higher COVID-19 mortality (Billingsley et al., 2020).

Between 60 and 80 years old, more men were diagnosed positively compared to women. The male disadvantage was particularly pronounced for the age group of the 65–70 years old, especially in Italy. A tentative explanation for the higher number of cases for men in Italy compared to other countries is that hospitalized patients were tested (especially at the beginning of the epidemic) and elderly men who were facing more serious consequences were more likely to be hospitalized. Although this is also the case for other countries (like France or the Netherlands), this pattern was more visible in Italy (Dudel et al., 2020). Other studies have confirmed this pattern (e.g. Gebhard et al., 2020).

While in absolute terms there were overall more women than men diagnosed with COVID-19 above 80 years of age, in relative terms—to the size of the total population—we observe that the male population above the age of 60 years was overrepresented compared to the female ones in the large majority of European countries. The COVID-19 related mortality risks are of particular concern for men as the case fatality rate is higher among men for all age groups. While this could largely depend on testing strategies, this finding was confirmed by the analysis of excess mortality, corroborating similar studies, not as exhaustive in terms of countries as the present one. For instance, a study of excess mortality in the first 4 to 7 weeks of the epidemic in England & Wales, France, Italy, the Netherlands and Portugal has revealed that excess deaths were in the range of 44%-107% for men, and 19–64% for women, the upper value being the one for Italy for both sexes (Félix-Cardoso et al., 2020).

The pandemic hit urban regions hardest in its first phase. Our results show that the virus evolved earlier—and with higher incidence—in urban regions, while we observe a slower evolution in intermediate and rural regions. The estimated R0 for the first weeks of the pandemic shows higher values in urban regions. During the course of the pandemic, the differences in R0 values across regions decrease, although the reproduction numbers remain above 1. The excess mortality associated with COVID-19 confirms the territorial differences in virus incidence during the first wave. Urban regions were disproportionately affected during the pandemic peak in April, recording median excess mortality values over 70% compared with previous years, and with mortality values approximately 18% higher for all months of the first wave. These findings support the results of previous empirical evidence on the role that population density and size have on virus transmission in the early phase of the pandemic. Through a cross-national test, we proved that population density intensifies virus transmission at the onset of the pandemic, as suggested by the analyses of Stier et al. (2020) and Ribeiro et al. (2020).

Our analysis faces some limitations because of the nature of the data collected at national and sub-national level. For the analysis based on the TESSy data, we are only able to consider diagnosed cases and fatalities. There are also significant differences in the number of tests and capacity of testing between countries and regions and over time. As a result, the completeness of case-based data varies by country (compared to the overall number of reported cases). This is why we added some analysis on excess mortality based on Eurostat data which confirmed the main findings based on cases and fatalities. A major limitation has to do with the fact the pandemic is still on-going and that therefore our analysis can only be considered valid for the first wave and not for the subsequent ones. The analysis shows the importance of accurate and timely collection of data that differentiate and categorize COVID-19 affected populations beyond the reporting of the sheer total number of cases and fatalities.

Overall, this paper shows the relevance of a demographic differentiated analysis of the COVID-19 infection in the European member states population, beyond health statistics, with the main idea to combat uncertainty. The observed significant heterogeneity by age, sex and place of residence of the population is a key information to guide the preparedness and response in healthcare facilities, the definition of policy interventions, particularly with the design of the preparedness strategy in view of the further spread of COVID-19 or other similar viruses. It shows that intervention and data collection planning should integrate socio-demographic and geographic profiling. The available information has already partially informed the gradual deconfinement and reconfinement plans during the second wave depending on the structure (e.g., prolonged and more sever confinement for residents and personnel in retirement houses), or on the region of residence (e.g. urban regions).

## Data Availability

Access to the data used for the analysis is restricted and cannot be made available.

## Footnotes

1 Density dependence refers to the effect of current and/or past population size on the per capita population growth rate.

2 Throughout the analysis, we use NUTS3 level regions. In some countries they correspond to administrative entities called provinces but this is not consistent across the European Union.

3 While the World Health Organisation (WHO) provides recommendations on testing all symptomatic individuals with fever, cough and/or difficulty breathing, screening procedures for COVID-19 that are adopted by national health authorities can vary between and within countries.

4 Metadata available from Eurostat: https://ec.europa.eu/eurostat/cache/metadata/en/demomwk_esms.htm (accessed 22/01/2021).

5 See for instance: https://www.ecdc.europa.eu/sites/default/files/documents/covid-19-rapid-risk-assessment-coronavirus-disease-2019-eighth-update-8-april-2020.pdf (May 24, 2020).

6 Among other initiatives, Euromod monitors the excess of mortality due to COVID-19 https://www.euromomo.eu/

7 https://www.npi.org.uk/blog/housing-and-homelessness/self-isolation-doesnt-work-crowded-households-government-needs-take-whos-advice-and-respond/ (blog accessed on 19/01/2021).

8 Eurostat's definition for urban-intermediate-rural regions is applied to NUTS level 3 regions. The urban–rural typology (https://ec.europa.eu/eurostat/web/rural-development/methodology) is a classification based on the following three categories: (a) predominantly urban regions, NUTS level 3 regions where more than 80% of the population lives in urban agglomerations; (b) intermediate regions, NUTS level 3 regions where more than 50% and up to 80% of the population live in urban agglomerations; (c) predominantly rural regions, NUTS level 3 regions where at least 50% of the population live in rural grid cells.

9 This method classifies metropolitan regions into 'large metro' and 'metro' regions based on the population size of the FUAs within the regions. TL3 non-metro regions are sub-classified into (a) with access to a metro, (b) with access to a small/medium city, or (c) remote based on their level of access to a FUA with population above a determined threshold. For more details, see https://www.oecd-ilibrary.org/urban-rural-and-regional-development/classifying-small-tl3-regions-based-on-metropolitan-population-low-density-and-remoteness_b902cc00-en (accessed 22/01/2021).
